# Supplementary material for: Effect of Sulfur Modification on Structural and Electrochemical Performance of Pitch-Based Carbon Materials for Lithium/Sodium Ion Batteries
Source: Nanomaterials (Basel). 2024 Aug 28;14(17):1410. doi: 10.3390/nano14171410 (PMC11397243; doi:10.3390/nano14171410)
Supplement: Supplementary file 1 [file nanomaterials-14-01410-s001.zip › nanomaterials-3106186-supplementary.pdf]

Effect of sulfur modification on structural and electrochemical performance of pitch-based carbon materials for lithium/sodium ion batteries

Zihui Ma [1†], Zhe Wen [1, 3†], Yan Song 1,2*, Tao Yang[1], Xiaodong Tian[1, 2], Jinru Wu [1], Yaxiong Liu[1],

Zhanjun Liu[1, 2] and Huiqi Wang[3*]

[1] CAS Key Laboratory of Carbon Materials, Institute of Coal Chemistry, Chinese Academy of Sciences, Taiyuan 030001, China; mazihui21@mails.ucas.ac.cn (Z.M.)
[2] University of the Chinese Academy of Sciences, Center of Materials Science and Optoelectronics Engineering, Beijing 100049, China
[3] College of Energy and Power Engineering, North University of China, Taiyuan, 030051, China
* Correspondence: yansong1026@126.com (Y.S.), hqiwang@nuc.edu.cn (H.W.)
† These authors contributed equally to this work.

Table S1. Elemental analysis results of PC and SPCs.

Samples	Elemental composition (%)					C/H[b]
	N	C	H	S	O[a]	
PC	1.00	93.48	4.12	0.58	0.82	1.89
SPC-4	0.96	87.03	3.44	7.81	0.76	2.11
SPC-8	0.89	80.59	2.61	15.30	0.61	2.57
SPC-12	0.81	78.33	2.72	17.42	0.72	2.40

Note. [a]Calculated by subtraction method. [b]Molar ratio.

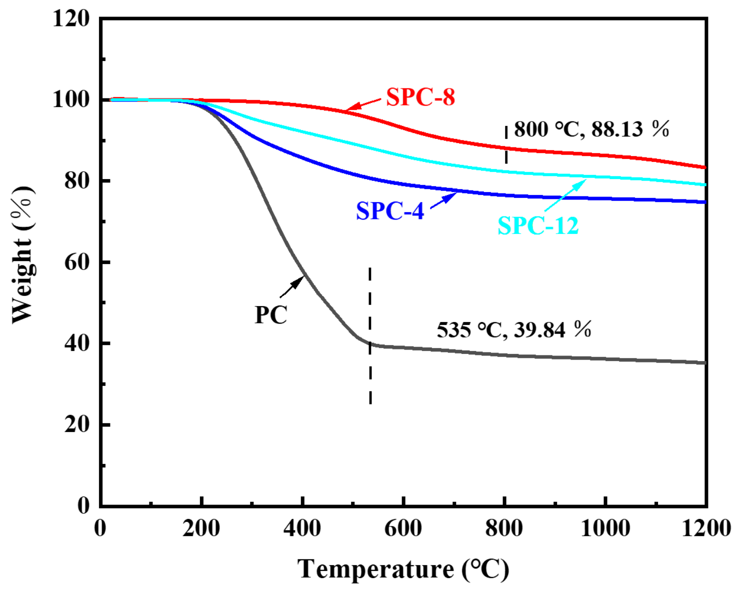

Figure S1. TG curves of PC and SPCs.

Comparing with the XRD data of standard graphite samples, we can calculate the layer spacing (d_{002}), the average width of aromatic sheet (L_a), and the average stacking height of aromatic sheet (L_c). Where d_{002} is the spacing between adjacent layers, L_a

is the average width of aromatic layers along the a-axis, and L_c is the average thickness of aromatic layers stacked along the c-axis. The size of L_a and L_c varies with the degree of polycondensation of pitch products. Generally, the greater the degree of polycondensation, the larger the values of L_a and L_c. The specific calculation formula is as follows:

Layer spacing: $d_{002} = \frac{\lambda}{2 \sin \theta_{002}}$ （Eq. S1）

Average width of aromatic sheet: $L_a = \frac{1.84\lambda}{\beta_{002} \cos \theta_{002}}$ （Eq. S2）

Average stacking height of aromatic sheet: $L_c = \frac{0.89\lambda}{\beta_{002} \cos \theta_{002}}$ （Eq. S3）

Of which, λ, β, θ_{002} is the wavelength of the incident X-ray, the half peak width of the diffraction peak and the (002) diffraction angle, respectively.

Table S2. Sheet structural parameters of PC-800 and SPC-800

Sample	$2\theta_{002}$	d_{002} (nm)	L_a(nm)	L_c(nm)
PC-800	25.8352°	0.353	1.1224	0.9229
SPC-800	25.4487°	0.358	1.1216	0.9222

The interlayer spacing of pitch products, PC-800 and SPC-800 gradually decreased from 0.353 nm to 0.358 nm. Furthermore, the values of L_a and L_c increased from 1.1224 nm and 0.9229 nm to 1.1216 nm and 0.9222 nm, respectively. This indicated that the addition of sulfur hindered the orderly growth of pitch molecules at high temperatures. Table S2 showed the lamellar structure parameters of PC-800 and SPC-800.

Table S3. Element content of PC-800 and SPC-800.

Samples	Elemental composition (%)				
	N	C	H	S	O
PC-800	0.92	86.23	5.98	0.23	6.64
SPC-800	0.78	81.68	2.80	6.89	7.85

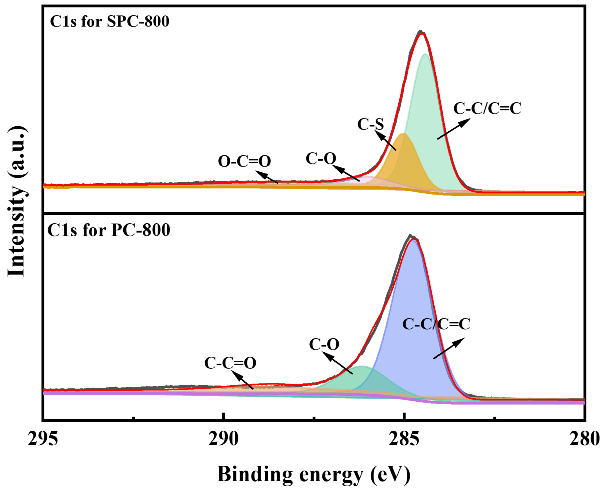

Figure S2. C 1s spectra of PC-800 and SPC-800.

Table S4. Comparison of electrochemical performance between SPC-800 and other reported anodes.

Samples	Capacity (mAh g^{-1})	Capacity retention	Rate capacity (mAh g^{-1})	Ref.
**SPC-800**	**478.1 (0.1 A g^{-1})**	**100% (100 cycles, 5 A g^{-1})**	**150.5 (5 A g^{-1})**	**This work**
**CSC-2**<br>Carbonized sulfur-doped porous carbon	**311 (0.1 A g^{-1})**	**-**	**146 (5 A g^{-1})**	**[32]**
**AC@G**<br>Amorphous sp^2+sp^3 carbon-coated sp^2 graphite	**434.1 (0.1 C)**	**96.8% (100 cycles, 6 C)**	**115.3 (5 C)**	**[33]**
**NiP+G**<br>Nanoparticle-doped pitch (NNDP) and natural graphite (NG)	**460 (20 mA g^{-1})**	**-**	**220 (1 A g^{-1})**	**[34]**
**BCG**<br>Graphitized of bituminous coal	**310 (0.1 A g^{-1})**	**95.3% (100 cycles, 0.2 C)**	**153.5 (5 A g^{-1})**	**[35]**
**BCNF**<br>Graphitized biogas-derived carbon nanofibers	**321 (0.1 C)**	**77% (50 cycles, 0.1 C)**	**50 (2 C)**	**[36]**

Note:1 C = 372 mA g^{-1}
